# Detection of rat lungworm (*Angiostrongylus cantonensis)* infection by real-time PCR from the peripheral blood of animals: a preliminary study

**DOI:** 10.1007/s00436-024-08251-9

**Published:** 2024-06-12

**Authors:** Susan Jarvi, John Jacob, Alfred Mina, Malia Lyons

**Affiliations:** 1 Department of Pharmaceutical Sciences, Daniel K. Inouye College of Pharmacy, University of Hawaii at Hilo, Hilo, HI USA; 2 Maikai Veterinary Clinic, Hilo, HI USA

**Keywords:** Rat lungworm, PCR, Blood, Diagnostics, *Angiostrongylus cantonensis*

## Abstract

Rat lungworm disease or neuroangiostrongyliasis is a cerebral parasitic infection that affects humans and animals alike. Its clinical signs and symptoms can range from mild self-resolving to serious life-threatening conditions. Studies suggest therapeutic interventions during the early stages of infection to be more effective than in later stages. However, early diagnosis of infection is usually problematic without the knowledge of exposure and/or detection of the parasite’s DNA or antibody against the parasite in the cerebrospinal fluid. This requires a lumbar puncture, which is an invasive procedure that generally requires hospitalization. This study evaluates an affordable and less invasive alternative to detect parasitic DNA by PCR from the peripheral blood of potentially infected animals. Blood samples from 58 animals (55 dogs and 3 cats) with clinical suspicion of infection were submitted to our lab between February 2019 and August 2022 by local, licensed veterinarians. DNA was extracted from whole blood, plasma, serum, and/or packed cells using the Qiagen DNeasy Blood & Tissue Kit as per the manufacturer’s protocol. All 58 animals were tested by real-time PCR using the AcanITS1 assay and 32 of these animals (31dogs; 1 cat) were also tested using the AcanR3990 assay. The PCR results for both assays were classified into strongly positive > positive > weakly positive > negative, and equivocal for ambiguous results, based on the strength of the signal. The percent infection detected using the AcanITS1 and AcanR3990 assays was 12.72% (7/55) and 20.68% (6/29), respectively. The overall percent infection detected was 34.37% (11/32), with only two animals testing positive by both assays. The three cats involved in this study tested negative by both assays. These results are promising and warrant further investigations to increase sensitivity including variables that might affect detection in the blood, such as parasite load, and laboratory methodologies.

## Introduction

*Angiostrongylus cantonensis* or rat lungworm is a zoonotic and neurotropic nematode. It is the causative agent for the clinical condition known as neuroangiostrongyliasis, which usually results in eosinophilic meningitis and associated complications. *Angiostrongylus cantonensis* has a complex life cycle that requires gastropods as the obligatory intermediate hosts and rats as definitive hosts (Graeff-Teixeira et al. 2009). The L3 stage of this parasite is infectious to animals and humans alike and is usually contracted through accidental or intentional ingestion of infected gastropods on food or grass (Wang et al. 2008). An infected slug or snail in a pet’s water dish could potentially release L3s, and consumption of this contaminated water could also possibly cause infection (Cheng and Alicata 1964; Howe et al. 2019). Reports also suggest the consumption of infected paratenic hosts to be another potential route of transmission (Wang et al. 2008; Niebuhr et al. 2020). The incubation period for neuroangiostrongyliasis is non-specific and can range from days to weeks. Clinical signs and symptoms can be non-specific and can range from mild and self-resolving to serious life-threatening conditions. The overall non-specific nature of the disease makes detection and diagnosis very challenging, especially without the knowledge of exposure (Wang et al. 2008; Barratt et al. 2016).

Currently, the State of Hawaii, particularly East Hawaii island, is reported to be the epicenter of neuroangiostrongyliasis in the USA, with over 24 human cases reported between 2001 and 2005 (Hochberg et al. 2007), and over 82 reported human cases between 2007 and 2017 (Johnston et al. 2019). These high rates of infection are thought to be due to the introduction of the invasive semi-slug species *Parmarion martensi*, which is known to be an extremely efficient intermediate host, capable of harboring large numbers of *A. cantonensis* L3s (Hollingsworth et al. 2007, 2013). Additionally, east Hawaii island receives heavy rainfall throughout the year keeping the environment damp and moist, making it ideal for the sustenance of any slug or snail species (Hollingsworth et al. 2007, 2013).

Studies suggest therapeutic interventions such as anthelmintic-corticosteroid treatments to be more effective during the early stages of infection than in their later stages (Ansdell et al. 2021; Jacob et al. 2022). Due to the non-specific nature of the early onset of the disease, diagnosis based on signs and symptoms is not definitive. The only other tool for diagnosis is by detection of the parasite’s DNA and/or antibodies from the cerebrospinal fluid (CSF) (Qvarnstrom et al. 2016). Collection of CSF requires a lumbar puncture (LP), which is an invasive procedure, with its own risks and complications. Some of the risks associated with LPs include prolonged back pain and headaches, stylet-associated complications, nerve root irritation, and spinal herniation (Evans 1998). Thus, a less invasive and risk-free alternative tool is desired.

In dogs infected with *Angiostrongylus vasorum* (dog heartworm), detection of parasitic DNA in peripheral blood using real-time PCR has been shown to substantially increase the detection rate, when combined with ELISA data (Jefferies et al. 2011). Similarly, a reliable assay to detect *A. cantonensis*’ presence in peripheral blood could provide valuable information to both veterinary and human clinicians. The potential for *A. cantonensis* DNA to be detected in the peripheral blood is an area of little inquiry. A study that challenged lab-reared wild rats with infective L3s demonstrated that *A. cantonensis* DNA could be detected in the peripheral blood of rats at 53 min, 1.5 h, 18 h, 5 weeks, and 6 weeks post-infection (Jarvi et al. 2015) and in a horse using the AcanR3990 assay (Sears et al. 2021). This study evaluates the capability of the already established PCR assays (AcanITS1 and AcanR3990) for detecting *A. cantonensis* DNA in the peripheral blood of potentially infected animals.

## Methodology

### Sample collection

A total of 58 animal blood samples (55 dogs and 3 cats) were submitted by Maikai Veterinary Clinic, Hilo, Hawaii, to confirm *A. cantonensis* infection by PCR (AcanITS1 and/or AcanR3990). These animals were either exhibiting clinical signs suspicious of *A. cantonensis* infection or had a known or suspicious history of consumption of a slug or a snail. These samples were submitted and tested between February 2019 and August 2022. Blood samples included EDTA-treated whole blood, serum, and whole clotted blood; plasma and lithium heparin blood and STRECK tube samples were also submitted. If SSTs (serum separator tubes) were submitted intact, the packed cells below the separator gel were tested in addition to and separately from the serum. All samples were refrigerated immediately after collection, transported on ice to the laboratory (except samples in STRECK tubes), and stored in the refrigerator until processing (DNA extraction and storage), and generally completed < 48 h from receipt of samples. A total of 100–200 μL of liquid sample or the equivalent volume of clotted blood was reserved for DNA extraction in 500 μL of DNA lysis buffer (0.1 M Tris–HCl, 0.1 M EDTA, and 2% SDS).

### Sample preparation

#### Serum and packed cells

The serum was spun by providers of samples and either transported in the original, intact SST, from which serum was decanted in a sterile biosafety cabinet, or serum was received separately from the provider in a sterile red-top glass tube. If the entire SST was submitted, the separator gel was removed with sterile implements and packed cells were retrieved for DNA extraction.

#### EDTA-treated blood

EDTA blood samples were collected in lavender top tubes and refrigerated until transport to the lab for processing where it was aliquoted into sterile 2.0-mL cryotubes.

#### Plasma

Plasma was either spun by the submitting providers or spun in-house from EDTA-treated blood submitted. Blood was spun in microcentrifuge tubes at 3000 RPM for 5 min and the supernatant was drawn off.

#### Whole blood

Whole clotted blood was collected without additives and ~ 100 µL was excised from the specimen with sterile implements in a biosafety cabinet.

#### *Lithium* heparin-treated blood

Lithium heparin blood samples were collected in Li Hep tubes and refrigerated until transport to the lab for processing. Once received, lithium heparin blood was aliquoted into sterile, 2.0-mL cryotubes.

#### STRECK tubes

Streck (La Vista, NE) manufactures proprietary cell-free DNA BCT® blood collection tubes (STRECK tubes) which contain K3EDTA (anticoagulant) and other DNA preservatives, enabling blood samples to be stored at room temperature for up to 14 days.

### DNA extraction

All extractions were performed using Qiagen DNEasy kit (Qiagen, MD USA) as per their blood protocol with modifications: 100–200 μL of blood product was used; a lower volume (100 μL) of elution buffer was used per elution to increase final concentration of DNA, and two elutions were performed with each sample to elute all DNA from the column. The two 100-μL elutions were stored separately, and for many samples, 9 μL of the first elution was used as the template for PCR reactions. DNA was stored at – 20 °C until PCR was performed.

### Real-time polymerase chain reaction (PCR)

All real-time PCR runs were performed using a Custom TaqMan Gene Expression Assay (Life Technologies, Grand Island, NY) on a StepOne Plus PCR thermocycler (Applied Biosystems, Foster City CA). Species-specific primers and probes based on the *A. cantonensis* AcanITS1 region and/or AcanR3990 (Qvarnstrom et al. 2010; Sears et al. 2021) were used along with 1X TaqMan Environmental Master Mix 2.0 (Life Technologies) as previously described (Jarvi et al. 2012, 2015; Howe et al. 2019) with the modification of increasing cycle number to 50 cycles of 94 °C for 30 s, 65 °C for 30 s, and 72 °C for 60 s (Niebuhr et al. 2020). Though the number of cycles was set to 50, the cut-off threshold for data reporting was 40 cycles (*C*_t_ ≤ 39.5). The additional 10 cycles were included to determine the nature of low-late amplifications, i.e., to observe if the low-late amplifications are exponential, achieving a plateau. Each sample, along with low- and high-concentration plasmid standards as positive controls, and a negative dH_2_O control were run in triplicate.

### Data analysis

The StepOne system software was used for analysis with the amplification threshold set manually at 0.25 fluorescence units for all reactions. The results from the PCR runs were categorized based on the strength of their amplification, (i.e., “strong positive” > “positive” > “weak positive” > “negative” and “equivocal” for ambiguous results) and based on the following ten criteria: I) All negative controls should be void of amplifications, and positive controls should be positive with a cut-off *C*_t_ ≤ 39.5. II) If 3 out of 3 triplicates amplify with *C*_t_ ≤ 35, the sample was considered as “strong positive.” III) If 2 out of 3 triplicates amplify with *C*_t_ ≤ 35, and the remaining 1 out of 3 amplify with a *C*_t_ > 35 (but less than or equal to 39.5), the sample was considered as “strong positive.” IV) If 1 out of 3 triplicates amplify with *C*_t_ ≤ 35, but the remaining 2 out of 3 triplicates amplify with *C*_t_ > 35 (but less than or equal to 39.5), the sample was considered as “positive.” V) If 2 out of 3 triplicates amplify with *C*_t_ ≤ 35, but the remaining 1 triplicate did not amplify, the sample was considered as “positive.” VI) If all the triplicates amplify with *C*_t_ > 35 (but less than or equal to 39.5), the sample was considered as “weak positive.” VII) If 2 out of 3 triplicates amplify with *C*_t_ > 35 (but less than or equal to 39.5), but the remaining 1 triplicate did not amplify, the sample was considered as “weak positive.” VIII) If 1 out of 3 triplicates amplify with a *C*_t_ ≤ 35, another 1 out of 3 triplicates amplify with a *C*_t_ > 35 (but less than or equal to 39.5), and the remaining triplicate did not amplify, the sample was considered as “weak positive.” IX) If only 1 out of 3 triplicates amplify, whether the *C*_t_ value is greater than, less than, or equal to 39.5, the sample was considered as “equivocal,” as these were ambiguous results. X) If none of the triplicates amplify, the sample was considered as “negative.” Similar criteria were used in previous studies (Baláž et al. 2023).

### Clinical data collection

Clinical information on animal patients was collected retrospectively from the clinic’s database. Clinical information such as clinical signs suspicious of *A. cantonensis* infection, behavioral changes in eating habits, walking, sitting, running, and tail movement (if applicable), medication/medical history, and potential source of infection were collected.

## Results

### Real-time PCR using AcanITS1 assay

All 58 animal blood samples (55 dogs and 3 cats) were tested using the same primers and probe (AcanITS1) currently used by the Hawaii Department of Health (HDOH) and by the Centers for Disease Control (CDC) (Qvarnstrom et al. 2016; Johnston et al. 2019) for detection of *A. cantonensis* DNA in the CSF as the definitive diagnostic for angiostrongyliasis in humans. The results show that 12.72% of the animals tested here (7/55 animals; excluding the 3 equivocal results) were positive using the AcanITS1 assay. The breakdown of results based on the strength of amplification is shown in Table 1.

**Table 1:** Categorization of *Angiostrongylus cantonensis* PCR results based on the strength of their amplification

Strength of amplification	Number of infected animals (AcanITS1)	Number of infected animals (AcanR3990)
Strong positive	1	0
Positive	2	1
Weak positive	4	5
Negative	48	23
Equivocal	3	3
Total	58	32
Capability (excluding equivocal)	7/55 (12.72%)	6/29 (20.68%)

### Real-time PCR using AcanR3990 assay

The AcanR3990 assay is a recently developed PCR method by the National Institute of Health (NIH). It is estimated to be ~ 1000 times more sensitive than the traditional AcanITS1 assay (Sears et al. 2021). Among these 58 animals, 32 (31 dogs and 1 cat) were also tested using the highly sensitive Acan3990 assay. The results show that 20.68% (6/29 animals; excluding the 3 equivocal results) of the animals were positive using the AcanR3990 assay. The breakdown of results based on the strength of amplification using the AcanR3990 assay is also shown in Table 1. Among the six animals that showed amplification, four animals were exclusive to the AcanR3990 assay (1 “positive” and 3 “weak positive” animals), and the remaining two animals showed amplification with both assays. Interestingly, both these animals were amplified as “positive” on the AcanITS1 assay but came as “weak positive” on the AcanR3990 assay, even though AcanR3990 has much higher sensitivity. All three cats tested in this study were negative by both assays. The combined percent infection detected among the animals tested using both assays was 34.37% (11/32 animals).

### Clinical correlations with PCR results

We were able to retrospectively retrieve the clinical information of 34 animals (32 dogs and 2 cats), among the 58 animals used in this study. Among these 34 animals, five dogs (14.7%) showed PCR amplification (2 by AcanITS1 and 5 by AcanR3990). Among these are the two dogs mentioned before that showed amplification by both assays. The clinical summary of these 34 animals and their association with PCR results are summarized in Table 2.

**Table 2:** The clinical signs suggestive of *A. cantonensis* infection, the frequency of their occurrence, and their association with PCR results

Clinical signs	No. of animals (*n* = 34)	No. of PCR-positive animals (*n* = 5)
Unable to sit, stand and/or run	25/34 (73.52%)	5/5 (100%)
Abnormal tail wagging	3/34 (8.82%)	0/5
Shaky or weak hind legs	11/34 (32.35%)	1/5 (20%)
Dragging hind legs	10/34 (29.41%)	2/5 (40%)
Hyperesthesia in the hind legs	4/34 (11.76%)	2/5 (40%)
Hyperesthesia of tail/tail base	6/34 (17.64%)	2/5 (40%)
Hyperesthesia across the spine	9/34 (26.47%)	1/5 (20%)
Kyphosis	3/34 (8.82%)	0/5
Paraplegia	4/34 (11.76%)	1/5 (20%)

The most common symptom was hind legs and/or tail base abnormalities, including in the five dogs that were positive by PCR amplification. Other studies have also reported hind leg and tail base abnormalities such as pain, hyperesthesia, and paresthesia to be common symptoms among *A. cantonensis* infections in animals, particularly in dogs (Lee et al. 2021; Baláž et al. 2023). Among these 34 animals, two (1 dog and 1 cat) were tested for *A. cantonensis* infection at the request of the pet’s owner, either due to the exhibition of suspicious signs or a history/suspicion of ingestion of slug(s). The remaining cat, even though was PCR negative by both assays, was exhibiting suspicious clinical signs such as difficulty walking, dragging the hind legs, paraplegia in the hind legs, and hyperesthesia in the thoracic-neck region.

All the animals (*n* = 34) received treatment for symptomatic relief such as analgesics; additionally, corticosteroids and anthelmintics such as fenbendazole and metronidazole (also an antibiotic) were given to most of the animals.

## Discussion

Most rat lungworm cases are due to the accidental ingestion of infected gastropods. Therefore, most patients are unaware of when or where they contracted the parasite. The source of infection in the dogs and cats in this study is unknown; however, one can presume an environmental source is likely involved in most cases. Slugs, snails, and rats are attracted to food and water sources provided for domestic animals. One of the most effective means of attracting slugs is dog food (Hollingsworth et al. 2007). Water sources are also a potential source of transmission (Howe et al. 2019). The early onset of symptoms is very general and non-specific in both humans and other animals (Barratt et al. 2016), thus making the diagnostic aspects of the disease complicated and often confusing. In dogs, the most common symptoms are associated with hind legs and/or tail base, such as pain, hyperesthesia, and paresthesia (Lee et al. 2021; Baláž et al. 2023). Similar symptoms were common among the dogs in this study, providing some degree of confidence that they might be infected with rat lungworm.

Currently, the only confirmatory method for the diagnosis of *A. cantonensis* infection is by either isolation of the parasite itself or by the detection of DNA/antibodies from the CSF. This requires the patient to undergo a lumbar puncture, also known as a spinal tap, which is an expensive and invasive procedure that has its own risks and complications (Evans 1998). This study investigated the capability of the established AcanITS1 and AcanR3990 Real-time PCR assays for detecting *A. cantonensis* DNA from the peripheral blood of potentially infected animals.

Detection of parasitic DNA by PCR in bodily fluids is a chance reaction and it is an attempt at finding the proverbial “needle in a haystack.” The infective L3 stage of this parasite is microscopic, and since *A. cantonensis* is not a blood parasite, their time residing within the peripheral bloodstream en route to the CNS (central nervous system) is finite, whereas they reside in the CNS for a longer period (approximately 7 days) (Mackerras and Sandars 1955; Bhaibulaya 1975). Additionally, while collecting blood samples from the animal, only a small quantity (< 10 mL) is collected for testing. It is again only chance as to whether the collected blood will, in fact, contain the parasitic DNA. Although the same can be argued while attempting to detect parasitic DNA from the CSF as well; the average volume of CSF in a healthy human is approximately 120 to 150 mL at any given time (Trevor Huff et al. 2023), and similarly, the average volume of blood circulating is approximately 5000 mL (Sharma and Sharma 2023). Considering this drastic difference in volumes and extended duration of parasites’ residence in the CNS, parasitic DNA is more likely detectable from the CSF than from the blood.

Nonetheless, DNA was detected in the peripheral blood of experimentally infected rats as early as 53 min and up to 6 weeks post-infection (Jarvi et al. 2015), using the AcanITS1 assay and in a horse using the AcanR3990 assay (Sears et al. 2021). Interestingly, a few past studies with experimentally infected rats (Jarvi et al. 2015; Mackerras & Sandars 1955) demonstrate that not all infective L3 develop into adults in a given rat and so, where do they go? These “lost larvae” may be a source of biological remnants of nematodes that contain genetic material that might potentially be found in peripheral blood for an indeterminate duration (Jarvi et al. 2015).

All blood samples were spun to obtain either plasma/serum and packed cells/clotted blood. The ability of both assays to detect parasitic DNA from the type of blood product was almost identical for both assays: EDTA blood > serum > whole clotted blood. This is an interesting trend that requires further validation to determine the choice of blood product that is most efficient for the detection of *A. cantonensis* DNA. In this study, we defined PCR runs as equivocal if only 1 out of 3 triplicates amplified regardless of whether the *C*_t_ value was greater than, less than, or equal to 39.5. All the equivocal samples were run multiple times with the intention of obtaining a definitive result. However, in the majority, the results kept repeating as equivocal. This may be due to the limited amount of parasitic DNA typically present within the collected samples which made detection sporadic using these PCR techniques.

Development and implementation of a blood-based PCR diagnostic method would allow rapid confirmation of infection, thereby allowing earlier initiation of rat lungworm-specific treatment. It is reported that pharmacological treatments respond better during the early stages of infection than in their later stages (Ansdell et al. 2021; Jacob et al. 2022). Since the animals enrolled in this study are based only on the suspicion of *A. cantonensis* infection (either by the veterinarian or the pet’s owner), the absolute infection rate among these animals cannot be estimated. At present, absolute *A. cantonensis* infection can only be confirmed by larvae/worm recovery from the animal (or human), which is not always possible and is a very rare opportunity (Graeff-Teixeira et al. 2009). Therefore, our study is unable to establish the sensitivity of AcanITS1 and AcanR3990 for detecting *A. cantonensis* DNA from the peripheral blood, but it indicates that such a method is effective and requires further optimization to increase its sensitivity.

## Data Availability

No datasets were generated or analysed during the current study.
